# Is a Crying Babe Necessarily Colicky

**Published:** 1867-04

**Authors:** 


### *Is a Crying Babe necessarily Colicky?*

Nothing is more common than the belief than when an infant cries it must have the colic, and that it should be treated accordingly. Now, can it be true that infants never cry unless they suffer pain, and that colic is the most common cause of this pain? Have we not, on the contrary, every reason to believe that the cry of the infant is merely a substitute for language, and is therefore used to make known to the mother or nurse such simple wants as may be experienced by one so young? While it would seem probable that an infant who suffers no pain, and who is sufficiently supplied with its natural food, can have no cause to cry, such is not always strictly the case. There is a great difference in the temper and disposition of infants; some being naturally irritable, cross or peevish, and others good-natured and cheerful. All nurses understand the difference between a good and a bad child; and it would be interesting to take note of these early indices, for the purpose of ascertaining whether or not they may be relied upon as the promonitions of subsequent developments in the adult. Some infants will remain quiet until a sense of hunger or thirst impels them to cry out; while others will cry to be turned over, or to be taken in the arms, or even to be walked about; and if these caprices are indulged, the child soon becomes so "spoiled" that its nurse will have no rest. It is surprising how soon the infant learns by experience what he may exact by his cries; and, although born good tempered, he may become extremely troublesome if too much indulged. Some of them only a week old will keep the nurse all the time busy, merely because they were not at first allowed to cry at all, without being handled.

It can not be denied that peevishness is, alike in infants and adults, very often consequent upon the discomfort of bad health; and it is important that the cries occasioned by this state of things be distinguished from those induced by actual pain. A judicious mother or nurse can not fail to discover the difference by a little careful observation, and it should be the duty of the medical adviser to assist in this diagnosis; for until the real cause of the cries be ascertained, there can be no rational medication. The cries of an infant are in reality only symptoms of the mental or physical condition of the child. It is our business to give to them their proper interpretation. The child cries! Is it caprice; is it hunger; is it discomfort; or is it positive pain? These are the questions to be solved before we should resort to medication, if we wish to be consistent with philosophy, or even with ordinary common sense. And yet, how often do we not find infants dosed with "*colic drops*" whenever they cry!

Most of the nostrums vended as "colic drops" contain opium in some form or other, and some aromatic or carminative. These "drops" are therefore primarily narcotic and stimulant, and secondarily constipating; so that, although they may compose or put the child to sleep, whether the cries proceed from colic or not, their use, or rather their abuse, is objectionable. Again, how are we to determine that the child has colic? Pain in the bowels may depend upon spasmodic contractions of their muscles induced by indigestion, or irritation of some kind; or it may be occasioned by mere flatulency. While the spasmodic pains usually precede or attend looseness of the bowels, such need not be the case with the presence of flatulency. The former pains come on in paroxysms more or less severe, which subside very soon, and leave the patient entirely relieved until they return again. Flatulent colic is more persistent, never so intense, and may be usually recognized by the hollow sound produced by percussion of the abdomen, especially if this circumstance be taken in connection with the other points in the history of the case.

The diagnosis of infantile diseases is by no means so difficult as is generally imagined. In the affection before us, it is just as easily made out for a child as for an adult. If the physician knows his business, and will use with due diligence the resources of art, he will rarely fail to establish the diagnosis satisfactorily.

If the bowels are regular and the evacuations in a natural state, while the abdomen yields a natural sound upon percussion, has a natural feel to the hand, is not distended nor knotted by spasmodic contractions, is not painful when pressed upon, we may very safely conclude that the child can not have colic.

Have we any good grounds to believe that colic is often almost habitual in infants too young to speak and who can only cry, whereas it is only an accidental or occasional disease in those who can speak and in adults? Such a violation of analogy ought not to be admitted to exist without much more evidence than can be adduced in favor of it.

Ear-ache is very common with children, and may either make them peevish or cause them to cry violently and protractedly. This affection can always be detected by pressing a finger just below or in front of the ear, by which the pain will be much increased and the child will renew his cries. As there is usually but one ear affected at the time, the experiment must be tried on both sides. If the pain be purely neuralgic or nervous, it may be relieved by almost any application; but if it be occasioned by the formation of an abscess about to break in the ear (in which case we may usually detect a little fulness or hardness in the angle just below the ear, or in the slight depression just in front of the orifice of the ear,) these remedies are very apt to fail, and we have to resort to a little Laudanam taken internally, or dropped into the ear in combination with a few drops of oil.

Closely connected with the treatment of the so-called colic is the common practice of

### *Jolting Infants.*

If the child be really suffering with colic, it would be as absurd to expect to relieve it by such violent shaking and jolting, as it is to suppose that there is any efficacy in the veterinary practice of making a colicky horse trot up and down the road until almost exhausted. But if the poor child happens to have pain in the ear, or head-ache, both of which are very common, the cruelty of violent rocking, shaking in the arms, and jostling upon the knees, with the loud singing and jargon of the nurse, must be apparent. The treatment of Sancho Panza by the maid of the enchanted castle was trifling in comparison with this.

The affectionate and tender-hearted mother can not bear to remain quiet while her babe is screaming, and she freely exerts her lungs and limbs to the uttermost in the hope of giving relief. It is a natural and a laudable feeling which prompts her, and the exertion relieves *her* nervous system by working off the nerve force which would have been otherwise concentrated in the brain. It therefore requires some philosophy, that which emenates from enlightened reason, to examine quietly for the true cause of the child's cries, and to administer the proper remedy. If no medicine be necessary, the child will, if laid comfortably on his bed, or held quietly in the mother's lap, very generally go to sleep after crying a little while. It can certainly not go to sleep so long as it is not allowed to be at rest.

### *Do Children bear Disease better than Adults?*

To suppose that children can bear disease better than adults, is to admit that the weak have more powers of resistance than the strong; that an unfinished fortification is better adapted to resist attacks than one already completed. And yet, we continually hear persons manifesting a desire that their children might take the measles, hooping-cough, etc., while young, so as to be rid of subsequent danger! This is a radical error. Children should be kept from sickness as long as possible, for no one can predict the result of what might at first seem to be the most trivial affection.

Common sense should lead us to avoid sickness at all times, and at any age. If we carefelly keep our children from visiting houses in which there is any sickness, and remove them from districts affected with epidemics; if, in short, we use due diligence in avoiding all known causes of sickness, we shall have nothing to reproach ourselves, when, notwithstanding such precautionary measures, they are overtaken by disease. The very fact that children are more prone to sickness than others, should incite parents to great watchfulness in regard to their hygienic condition, their cleanliness, their clothing, their food, their exercise, their supply of fresh air, insolation, etc., etc.

The best evidence that children do not bear sickness as well as adults, is to be found in our mortuary statistics, which reveal a frightful loss of life among infants and children. This is equally true with regard to the lower animals and plants. The more tender the plant the more feeble are its powers of resistance, and the more liable it is to disease. —*Southern Med. and Surg. Journal.* L. A. D.

